# A detailed phenotypic analysis of immune cell populations in the bronchoalveolar lavage fluid of atopic asthmatics after segmental allergen challenge

**DOI:** 10.1186/1710-1492-9-37

**Published:** 2013-09-17

**Authors:** Jonathan S Boomer, Amit D Parulekar, Brenda M Patterson, Huiqing Yin-Declue, Christine M Deppong, Seth Crockford, Nizar N Jarjour, Mario Castro, Jonathan M Green

**Affiliations:** 1 Department of Internal Medicine, Washington University School of Medicine, St Louis, MO 63110, USA; 2 Current affiliation: Department of Internal Medicine, Baylor College of Medicine, Houston, TX 77030, USA; 3 Department of Internal Medicine, University of Wisconsin School of Medicine and Public Health, Madison, WI 53792, USA

**Keywords:** T lymphocyte, CD30 expression, Segmental allergen challenge, Asthma

## Abstract

**Background:**

Atopic asthma is characterized by intermittent exacerbations triggered by exposure to allergen. Exacerbations are characterized by an acute inflammatory reaction in the airways, with recruitment of both innate and adaptive immune cells. These cell populations as well as soluble factors are critical for initiating and controlling the inflammatory processes in allergic asthma. Detailed data on the numbers and types of cells recruited following allergen challenge is lacking. In this paper we present an extensive phenotypic analysis of the inflammatory cell infiltrate present in the bronchoalveolar lavage (BAL) fluid following bronchoscopically directed allergen challenge in mild atopic asthmatics.

**Methods:**

A re-analysis of pooled data obtained prior to intervention in our randomized, placebo controlled, double blinded study (costimulation inhibition in asthma trial [CIA]) was performed. Twenty-four subjects underwent bronchoscopically directed segmental allergen challenge followed by BAL collection 48 hours later. The BAL fluid was analyzed by multi-color flow cytometry for immune cell populations and multi-plex ELISA for cytokine detection.

**Results:**

Allergen instillation induced pro-inflammatory cytokines (IL-6) and immune modulating cytokines (IL-2, IFN-γ, and IL-10) along with an increase in lymphocytes and suppressor cells (Tregs and MDSC). Interestingly, membrane expression of CD30 was identified on lymphocytes, especially Tregs, but not eosinophils. Soluble CD30 was also detected in the BAL fluid after allergen challenge in adult atopic asthmatics.

**Conclusions:**

After segmental allergen challenge of adult atopic asthmatics, cell types associated with a pro-inflammatory as well as an anti-inflammatory response are detected within the BAL fluid of the lung.

## Background

Asthma is a complex immunological disease affecting approximately 8-10% of the population of the United States [1-3]. The pathology of asthma includes pulmonary inflammation, airway eosinophilia, mucus hypersecretion and airway hyperreactivity (AHR), induced by specific and nonspecific stimuli which lead to an inappropriate Th2 response [1-3]. T lymphocytes of a Th2 type secrete IL-3, IL-4, IL-5, IL-9, and IL-13 [1-3], cytokines which play important roles in Th2 lymphocyte survival, B cell isotype switching to IgE and mast cell, basophil and eosinophil differentiation and survival [1-4]. This complex inflammatory cascade is controlled by soluble factors as well as interactions between cell types involving surface receptors such as CD80/86 on dendritic cells (DC) and CD28/CTLA-4 on T cells [5-9], ultimately resulting in the asthma phenotype [1-3].

Although inflammation is critical to the pathogenesis of asthma, the exact cell types present in the lung and their differentiation state remain not well defined. In this report, we characterized the immune cell populations and cytokines present within the lungs of mild atopic asthmatics that underwent bronchoscopy with bronchoalveolar lavage (BAL) after segmental allergen challenge (SAC). We detected an increase in pro-inflammatory cytokines (IL-6) and immune modulating cytokines (IL-2, IFN-γ and IL-10) along with an increase in lymphocytes and the suppressor cells, regulatory T (Treg) and myeloid derived suppressor cells (MDSC). Although we did not detect expression of CD30 on eosinophils, we did detect significant membrane expression of CD30 on Tregs and the presence of sCD30 in the BAL fluid of adult atopic asthmatics.

## Methods

Data collected from the Costimulation Inhibition in Asthma (CIA) trial (ClinicalTrials.gov NCT00784459) [10] was pooled and re-analyzed for this manuscript. For detailed methods and a description of the participant population and inclusion/exclusion criteria, please refer to reference [10]. In brief, nonsmoking males and females between 18 and 50 years of age with previously diagnosed mild asthma by NAEPP guidelines were identified. At screening, participants underwent skin prick testing of cat allergen extract, short ragweed allergen extract *(Ambrosia artemisiifolia*), and standardized dust mite allergen extracts (*Dermatophagoides farinae* and/or *Dermatophagoides pteronyssinus*) (all from Greer Laboratories, Lenoir NC) to determine their reactivity using standard methods [10-13] (Table 1). Eligible participants underwent bronchoscopy with segmental allergen challenge (SAC) with instillation of 5 ml allergen solution at a concentration 1000X the minimum skin reactive test dose, with a maximum of 1:100 dilution of the stock allergen . Repeat bronchoscopy was performed 48 hours later with BAL performed in the same sub-segment in which allergen had previously been instilled. The study was approved by the Washington University Institutional Review Board.

**Table 1:** Allergens used for titrated skin prick testing

	**1:100000**	**1:1000000**	**1:10000000**	**Total**
Cat	2	3	5	10
Ragweed	4	5	2	11
D farinae	1	0	1	2
D Pteronyssinus	1	0	0	1

The dilution of allergen that provoked a positive test during titrated skin prick testing is shown. Shown is the number of subjects that had a positive test at the indicated dilution of allergen. 24 subjects were recruited for the initial trial with pre-randomization data re-analyzed for this study. Adapted from Table E8 (reference [10]).

### Measurement of BAL total cell counts and differentials

In the research laboratory, total cell counts were determined, cytospins prepared and manual differentials performed immediately following bronchoscopy [10]. The BAL fluid was centrifuged and the supernatant concentrated, using Ultracentrifugation Filter Units (Millipore, Billerica MA), to approximately 1/20^th^ of the original volume, aliquoted and frozen at −80°C for further analysis.

### Flow cytometric analysis

The cell pellet recovered from the BAL was resuspended in PBS containing 1% BSA and incubated with antibodies directed against the following cell subsets: T cells were identified as CD4 or CD8, as naïve (CD45RA) or memory (CD45RO), and surface phenotyped for CD28, CD25 or CD30. B cells were identified as CD19+. NK cells were identified as CD56+. Regulatory T cells were identified by staining with CD4, CD25 and FoxP3. Dendritic cells were identified via lineage cocktail negative (CD3/CD14/CD16/CD19/CD20/CD56), HLA-DR high and either CD123+ for plasmacytoid dendritic cells (pDC), or CD11c+ for myeloid dendritic cells (mDC). Myeloid derived suppressor cells (MDSC) were identified as lineage cocktail negative, CD33+ and HLA-DR low. Eosinophils were identified by light scatter properties, CD16 negative and CD11b+. Fluorescently conjugated antibodies were purchased from BD Biosciences (San Jose, CA), BioLegend (San Diego, CA) or eBiosciences (San Diego, CA). In brief, 1-2x10^6^ BAL cells were labeled with fluorescently conjugated antibodies at room temperature for 30 minutes. After labeling, RBC were lysed in 1X RBC lysis buffer (eBioscience) for 1–2 minutes and extensively washed in FACS wash (eBioscience). For FoxP3 staining, after surface labeling, cells were labeled with anti-FoxP3 antibody according to the manufactures protocol (human FoxP3 kit, eBioscience). The labeled cells were then analyzed on a 4-color FACSCalibur flow cytometer using CellQuest software (Becton-Dickinson Corporation, Mountainview, CA). Samples were gated on a lymphocyte gate followed by a second gate relevant for the population being analyzed (i.e., CD4+) and a minimum of 10,000 gated events collected. Data was further analyzed using Winlist v7 software (Verity Software Corporation, Topsham, ME).

### Measurement of cytokine and soluble CD30 levels

BAL fluid was analyzed for cytokine content using the Cytokine Bead Array (Th1/Th2/Th17 kit, BD Biosciences) per the manufacturer’s instructions. The soluble fragment of CD30 (sCD30) was measured by a standard enzyme-linked immunosorbent assay (ELISA) per the manufacturer’s directions (human sCD30 ELISA kit, Abnova, Walnut, CA). The limit of detection of the CBA is 5 pg/mL while the sCD30 ELISA is 0.3 ng/mL.

### Statistical analysis

All data were analyzed using either SAS version 9.3 (SAS Institute, Cary, NC) or Prism version 4 (GraphPad Software Inc., La Jolla, CA). Outcomes are presented as the mean ± standard deviation or graphically as individual subject points. A non-parametric two-tailed Mann–Whitney U test was performed between pre- and post-SAC data. A 1-way ANOVA (Kruskal-Wallis) and Dunn’s Multiple Comparison Test for individual means was performed when analyzing groups of 3 or more. A p-value <0.05 was considered significant.

## Results

### Inflammatory response to allergen

**Figure 1:**
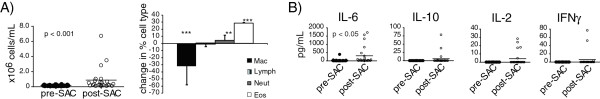
**Segmental allergen challenge (SAC) induces an inflammatory response detected in the BAL fluid of atopic asthmatics.** In **A)**, total cell counts and differential analysis are presented for BAL fluid obtained pre- and post-SAC. In **B)**, cytokine levels in the BAL fluid were measured by multiplex ELISA using the Cytokine Bead Array (CBA). Shown is the re-analysis performed on the 24 subjects enrolled in the randomized, placebo controlled, double blinded Costimulation Inhibition of Asthma (CIA) trial at pre-randomization. In **A** (right panel), the average (change in percent cell type) is presented with error bars representing ± the standard deviation with an (* = p < 0.05; ** = p <0.01; *** = p < 0.001). Each subject is represented by a single circle in the graphs with p values presented. Due to pre-SAC cytokines being below the detection limit of the assay (5 pg/mL), no statistical analysis was performed for IL-10, IL-2 or IFN-γ.

We re-analyzed data from 24 atopic asthmatics that underwent a bronchoscopy and allergen challenge prior to randomization in the parent trial [10]. As indicated in Figure 1, allergen challenge led to an increase in the total cell number present in the BAL fluid (1.5x10^5^ ± 6.5x10^4^ vs 8.8x10^5^ ± 1.5x10^6^; p < 0.001). There was also a significant change in the cellular composition, with an increase in both eosinophils (28.7% ± 26.2%) and neutrophils (4.5% ± 7.3%), along with a decrease in macrophages detected (−32.1% ± 26.0%) (Figure 1).

We measured the concentrations of pro-inflammatory (IL-6 and TNF) and T cell-derived (IL-10, IL-2, IL-17, IL-4 and IFN-γ) cytokines in the BAL by multi-plex ELISA. IL-6, was significantly induced following allergen challenge (30.8 ± 82.1 vs 311.6 ± 540.0 pg/mL, Figure 1B) while TNF was only detected in a single subject (< 10 pg/mL, data not shown). The T cell-dependent cytokines were not detected pre-allergen; however, IL-2 (4.2 ± 8.8 pg/mL), IFN-γ (6.0 ± 19.3 pg/mL) and IL-10 (5.9 ± 18.2 pg/mL) were detected in some of the subjects (Figure 1B). Neither IL-4 nor IL-17A was observed post-allergen challenge in BAL fluid (data not shown). Surprisingly, most subjects had no detectable levels of T cell-derived cytokines even after allergen instillation in the lung (Figure 1B). An analysis based upon the type of allergen instilled and/or the dilution of allergen that induced a positive skin test did not yield statistically relevant differences due to the limited number of subjects in each group (Table 1). These data indicate that the instillation of allergen in the lung induces the infiltration of inflammatory cells and the production of both pro-inflammatory and T-cell dependent cytokines.

### Allergen challenge results in recruitment of innate and adaptive immune cells to the lung

**Figure 2:**
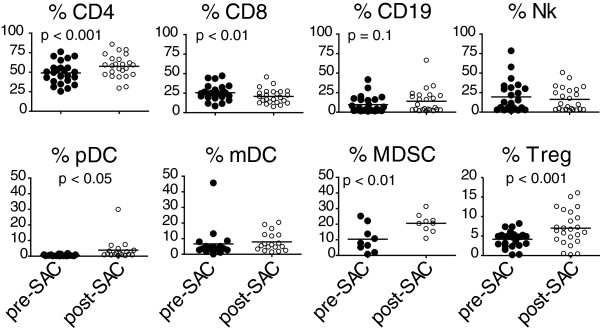
**Innate and adaptive immune cells are recruited to the BAL fluid after allergen challenge.** Cellular populations in the BAL fluid pre- and post-SAC were labeled with specific fluorophore conjugated antibodies and analyzed by multi-color flow cytometry. The percentages were calculated as a percent of the lymphocyte gate, with the exception of the DC (plasmacytoid [pDC], myeloid [mDC]) and myeloid derived suppressor cell (MDSC) subsets which were calculated as a percentage of the lineage cocktail negative gate. Each subject is represented by a single symbol on the graphs, with p values indicated.

Multi-color flow cytometry was utilized to provide a more detailed phenotypic analysis of immune cells recovered following allergen challenge. An increase in CD4+ T cells along with a decrease in CD8+ T cells was observed 48 hours following allergen challenge (Figure 2). The innate immune response to allergen has also been recognized as an important component of asthma [5]; therefore, we determined the recruitment of natural killer cells (Nk) and dendritic cells (DC) to the lung. No change in Nk or myeloid dendritic cells (mDC) was observed; however, a small yet significant increase in plasmacytoid dendritic cells (pDC; 0.5% ± 0.5% vs 3.9% ± 6.8%; p < 0.05) was observed in the BAL fluid after allergen challenge (Figure 2).

To further characterize the inflammatory response in the lung following allergen challenge, we enumerated the number of suppressor cells present in the lung. Regulatory T cells (Tregs) and myeloid derived suppressor cells (MDSC) are important mechanisms for down-modulating immune responses to antigen; interestingly, both were recruited to the lung following allergen challenge (Tregs: 4.2% ± 2.1% vs 7.0% ± 4.4% [p < 0.001]; MDSCs: 10.4% ± 8.5% vs 20.6% ± 5.9% [p < 0.01]) (Figure 2). In addition to being present in the BAL, this MDSC population expressed co-stimulatory B7-molecules (CD80 12.2% ± 17.4% and CD86 25.7% ± 12.5%, Additional file 1).

**Figure 3:**
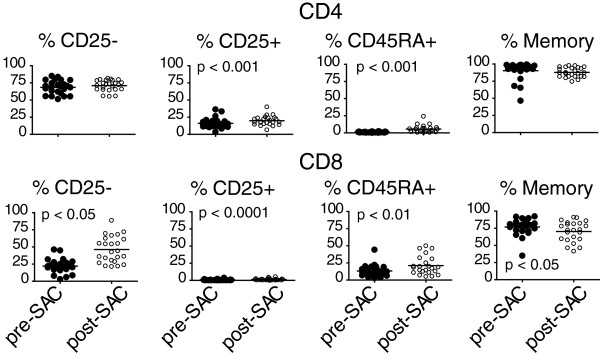
**Expansion in both naïve and activated T cells in the BAL fluid after allergen challenge.** T cell subsets in the BAL fluid pre- and post-SAC were determined by the expression of CD25 (IL-2R; activated), CD45RA (naïve or effector memory) and CD45RO (memory) by multi-color flow cytometry. The percentages were calculated as a percent of either the CD4+ or CD8+ T cell population. Each subject is represented as a single symbol on the graphs, with p values indicated.

In addition to analyzing the percentage of lymphocyte populations, we performed an extensive phenotypic analysis of these cell subsets. An increase in the percentage of CD45RA+ (naïve or effector memory) T cells (1.1% ± 0.6% vs 5.6% ± 5.4%; p < 0.001) CD4+ and CD8+ (13.3% ± 8.7% vs 21.2% ± 13.5%; p < 0.01) T cells was observed post-allergen challenge along with a reduction in memory (76.8% ± 12.4% vs 70.3% ± 14.8%; p < 0.05) CD8+ T cells (Figure 3). We detected an increase in the percentage of cells that expressed the activation marker CD25 (IL-2R) for both CD4+ (16.0% ± 7.4% vs 19.8% ± 6.9%; p < 0.001) and CD8+ (0.7% ± 0.8% vs 1.3% ± 1.3%; p < 0.0001) T cells (Figure 3). These data demonstrate that allergen challenge results in the recruitment of activated CD4+ and CD8+ T cells and innate immune cells along with specific regulatory cell populations.

### CD30 expression in BAL fluid after allergen challenge

**Figure 4:**
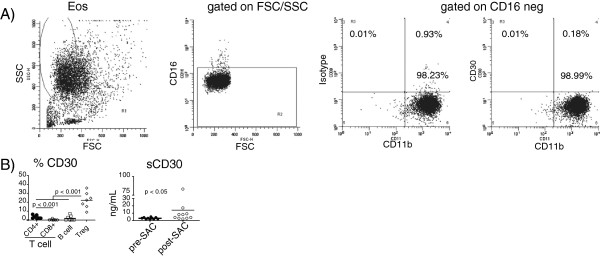
**CD30 expression is detected after allergen challenge in the BAL fluid.** In **A)**, eosinophils were analyzed for CD30 expression by flow cytometry by gating on high side scatter (left panel) followed by gating on CD16 negative cells with eosinophils determined as CD11b+. Shown are flow cytometry plots for a representative subject with CD30 expression on eosinophils compared to an isotype control (**A**, right 2 panels). In **B)** (left panel), membrane CD30 expression was analyzed on lymphocyte populations (CD4+ and CD8+ T cells ● and ○; B cells □ and Tregs ◊) post-SAC on 7 subjects by multi-color flow cytometry, with p-values determined by 1-way ANOVA (Kruskal-Wallis) with Dunn’s Multiple Comparison Test for individual means indicated. In **B)** (right panel), sCD30 was measured in the pre- and post-SAC BAL of 10 subjects by ELISA, with the p-value indicated.

CD30 has been shown to be expressed on eosinophils [14,15], and is associated with Th2 type T lymphocytes [16] and may correlate with severity of asthma [17,18]; therefore, we analyzed whether CD30 was expressed on BAL cells and present in the BAL fluid after allergen challenge. Contrary to some reports in the literature [14,15], we were unable to detect any CD30 expression on the surface of BAL eosinophils by multi-color flow cytometry (Figure 4) or by immuno-histochemical staining of cytospins (data not shown) either pre- or post-allergen challenge. However, CD30 expression was detected on a low yet significant percentage of T and B cells (Figure 4) and by immuno-histochemical staining of BAL cytospins (data not shown) obtained following allergen challenge. Of note, the percentage of CD30+ Tregs (22.1% ± 9.1%) was ~10-fold more than detected on either B cells (2.1% ± 2.8%) or T cells (CD4+ 3.2% ± 2.0%; CD8+ 0.3% ± 0.7%) (Figure 4). Soluble CD30 (sCD30), the cleaved fragment of membrane CD30 [19,20], was quantitated by ELISA in the concentrated BAL fluid. There was a 4-fold increase in sCD30 protein in the BAL fluid upon allergen challenge (3.0 ± 1.1 vs 14.1 ± 24.6 in pg/mL; p < 0.05) (Figure 4). These data identify the presence of CD30 positive lymphocytes, especially Tregs, but not eosinophils, and sCD30 in the BAL fluid after allergen challenge of atopic asthmatics.

## Discussion

In this study, we took advantage of results originally obtained in an interventional trial designed to test the effects of inhibition of T cell costimulation on the response of mild atopic asthmatics to allergen challenge [10]. Data obtained prior to administration of study drug provided a comprehensive data set describing the inflammatory response to allergen. We found that allergen instillation in the airway results in the recruitment of both innate and adaptive immune cells to the lung, including populations of suppressor cells. We also detected elevated expression of CD30, predominantly on regulatory T cells. Thus, the inflammatory response to allergen is a complex mixture of cell types.

Clinical and animal studies have supported the role of T lymphocytes as controllers of the aberrant Th2 initiated response and eosinophils as predominant effectors that induce asthma symptoms. The most dominant Th2 cytokine in asthma is IL-4 which has a plethora of activity including increasing IgE production, and differentiating T cells from a Th0 to Th2 [21,22]. We detected an increase in eosinophil percentage yet no significant increase in IL-4 after allergen challenge. Batra et al. determined the peak level of IL-4 is reached within 24 hours after allergen challenge [23] potentially explaining the lack of IL-4 detected in our study. In most subjects the majority of cytokines were below the limits of detection. This might reflect the extensive dilution that occurs due to the BAL procedure. Given this limitation, a meaningful biological difference is hard to conclude from these data.

Asthma is a predominate Th2 disease but there is also data for Th1 and Th17 responses. Th2 effector responses in asthma such as eosinophil recruitment, mucus production and AHR are inhibited by IFN-γ which is produced by Th1 T lymphocytes [1]. We detected a significant increase in IFN-γ as well as neutrophils which are recruited by IFN-γ [1] after allergen challenge (Figure 1A and 1B). Thus, our data supports a role for both Th2 and IFN-γ responses in asthma.

Allergen challenge in asthmatics induces changes in both innate and adaptive cellular populations. Although we did not detect increases in B lymphocytes, a significant increase in CD4+ T lymphocytes while a significant decrease in CD8+ T lymphocytes were observed (Figure 2). Furthermore, we observed alterations in T lymphocyte differentiation including: increases in naïve or effector memory (CD45RA+) percentages, increased CD25 (IL-2R) expression, and a decreased memory CD8+ pool after allergen challenge signifying a T cell response to antigen [1,24]. This is consistent with mouse models of asthma where increased levels of IFN-γ inhibit the generation of memory T cells [25] further contributing to the expansion of an effector T cell pool [26].

We did not observe differences in Nk cells or myeloid dendritic cells (mDC), innate cells important in asthma pathology [5]; however, a significant increase in plasmacytoid dendritic cells (pDC) was detected after allergen challenge. The recruitment of pDC may be part of the anti-inflammatory response in asthma [5,27]. In accordance with this anti-inflammatory response, an increase in regulatory T cells (Treg) as well as increased IL-10 secretion, a regulatory T and pDC effector cytokine, were detected after allergen challenge (Figures 2 and 1B). Regulatory T cells inhibit allergic responses by 1) suppressing myeloid dendritic cells (mDC) important for T lymphocyte activation and differentiation, 2) inhibiting Th1/Th2 and Th17 differentiation, 3) inhibiting IgE production, 4) preventing T lymphocyte migration into the lung and 5) inhibiting effector cell function of mast cells, basophils and eosinophils [5]. Importantly, we further identified an additional innate cell population termed myeloid derived suppressor cells (MDSC) as being increased after allergen challenge in adult atopic asthmatics (Figure 2). Our report is the first to identify MDSC in the BAL fluid of adult atopic asthmatics after allergen challenge. Interestingly in a mouse model of asthma, MDSC were shown to recruit regulatory T cells and down modulate T lymphocyte responses [28]. We also show that MDSC expressed co-stimulatory ligands, both CD80 and CD86, which have been associated with inducing Th2 T cells [3] as well as producing IL-10 [29]. Therefore, multiple suppressor cell types are associated with the anti-inflammatory response detected in the lung after allergen challenge.

Soluble CD30 is increased in the serum of pediatric asthma patients when compared to healthy individuals [18,30] as well as after allergen challenge in our study. We also detected sCD30 in the serum prior to allergen challenge (20.3 ± 8.0 ng/mL; data not shown) in adult atopic asthmatics. The role of soluble CD30 in asthma is not clear but is thought to block CD30-CD153 induced apoptosis and the induction of IgG class switching on B cells [31]. The presence of sCD30 in the BAL after allergen challenge may play a role in either preventing apoptosis of activated lymphocytes or in their differentiation. However, the role of sCD30 in asthma is unknown and requires further study.

Contrary to reports that detected mRNA and low surface expression of CD30 on eosinophils [14,15], we did not observe any CD30 expression on eosinophils by either flow cytometry (Figure 4A) or immuno-histochemistry (data not shown). We did however detect membrane CD30 on lymphocytes, especially regulatory T cells, after allergen challenge. CD30 expression on CD4+ T cells has been associated with IL-4 secretion [16] while CD30+ eosinophils undergo apoptosis in asthmatics [14,15]. Memory CD8+ T lymphocytes express CD30 [32], yet in our study the lowest percentage of CD30+ cells were CD8+ T lymphocytes. This may be partly explained by the decreased memory pool of CD8+ T cells after allergen challenge (Figure 3). There are no reports of CD30 expression on regulatory T cells in human asthma. In mouse model systems of allograft tolerance, a Th2 dominated response like asthma, CD30-deficient Tregs failed to reject grafts [33,34]. Due to the low cellularity of pre-allergen BAL sample, we were unable to measure CD30 expression on lymphocytes in most subjects. However, in a single subject, the percentage of CD30+ Tregs in the BAL after allergen challenge increased from 0.04% pre-allergen to 24.6% post-allergen challenge (data not shown). The role of CD30 expression on Tregs in asthma remains unclear; however, interaction between CD30 on the Treg and CD153 on a CD8+ T cell results in apoptosis of the CD8+ T cell [33,34]. Therefore, we speculate that CD30+ regulatory T cells and sCD30 play a role in controlling asthma in a CD30-dependent manner.

## Conclusions

Atopic asthmatics increase innate and adaptive immune cell populations after allergen challenge. In particular, alterations in both the activation and memory state of T lymphocytes as well as detection of a novel suppressor cell population termed MDSC. Furthermore, we detected soluble CD30 and identified significant expression of CD30 on immune cells, most notably on regulatory T cells, not eosinophils in atopic asthmatics after allergen challenge.

## Abbreviations

MDSC: Myeloid derived suppressor cells; BAL: Bronchoalveolar lavage; sCD30: Soluble CD30; SAC: Segmental allergen challenge; Th2: T helper type 2; Th1: T helper type 1; mDC: Myeloid dendritic cell; pDC: Plasmacytoid dendritic cell; Treg: Regulatory T cell; CIA: Costimulation inhibition of asthma trial.

## Competing interests

The authors declare that they have no competing interests.

## Authors’ contributions

JSB developed and performed the research assays and contributed to the formulation of the manuscript. ADP enrolled subjects, performed SAC protocols, analyzed specimens and data. HYD CMP and SC performed research assays and analyzed data. BMP was the lead research coordinator and was involved in all aspects involving participants. NNJ was the lead investigator at University of Wisconsin, MC and JMG collaboratively designed the study as well as provided oversight over the conduct of the entire study. All authors reviewed and contributed to the writing of the manuscript. All authors read and approved the final manuscript.

## Supplementary Material

Additional file 1 **Expression of co-stimulatory ligands (B7) on MDSC in the BAL fluid of atopic asthmatics.** Myeloid derived suppressor cells (MDSC) in the BAL fluid post-SAC were labeled with specific fluorophore conjugated antibodies and analyzed by multi-color flow cytometry. MDSC were identified by lineage cocktail negative, HLA-DR low and CD33+. The percent expression of co-stimulatory ligand expression on MDSC was analyzed by gating on MDSC followed by analysis of B7-1 (CD80) and B7-2 (CD86). Each subject is represented by a single symbol on the graphs, with p values indicated. Click here for file
