# Supplementary figures and images for: A detailed phenotypic analysis of immune cell populations in the bronchoalveolar lavage fluid of atopic asthmatics after segmental allergen challenge
Source: Allergy Asthma Clin Immunol. 2013 Sep 17;9(1):37. doi: 10.1186/1710-1492-9-37 (PMC3848528; doi:10.1186/1710-1492-9-37)

Myeloid derived suppressor cells  
(MDSC)

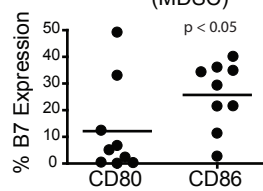

Supplement: Additional file 1 — Expression of co-stimulatory ligands (B7) on MDSC in the BAL fluid of atopic asthmatics. Myeloid derived suppressor cells (MDSC) in the BAL fluid post-SAC were labeled with specific fluorophore conjugated antibodies and analyzed by multi-color flow cytometry. MDSC were identified by lineage cocktail negative, HLA-DR low and CD33+. The percent expression of co-stimulatory ligand expression on MDSC was analyzed by gating on MDSC followed by analysis of B7-1 (CD80) and B7-2 (CD86). Each subject is represented by a single symbol on the graphs, with p values indicated. [file 1710-1492-9-37-S1.pdf]
